# Improving quality of care for cancer patients through oncological second opinions in a Comprehensive Cancer Center: adherence to second-opinion therapy recommendations

**DOI:** 10.1007/s00432-025-06149-2

**Published:** 2025-04-02

**Authors:** Carla Schulmeyer, Peter A. Fasching, Matthias W. Beckmann, Lothar Häberle, Henriette Golcher, Peter J. Goebell, Patrik Pöschke, Julius Emons

**Affiliations:** 1 Department of Gynecology and Obstetrics, Erlangen University Hospital, Comprehensive Cancer Center Erlangen-EMN, Friedrich Alexander University of Erlangen–Nuremberg, Universitaetsstrasse 21–23, 91054 Erlangen, Germany; 2 Biostatistics Unit, Department of Gynecology and Obstetrics, Comprehensive Cancer Center Erlangen-EMN, Friedrich Alexander University of Erlangen–Nuremberg, Erlangen, Germany; 3 Department of Surgery, Erlangen University Hospital, Comprehensive Cancer Center Erlangen-EMN, Friedrich Alexander University of Erlangen–Nuremberg, Erlangen, Germany; 4 Department of Urology, Erlangen University Hospital, Comprehensive Cancer Center Erlangen-EMN, Friedrich Alexander University of Erlangen–Nuremberg, Erlangen, Germany

**Keywords:** Second medical opinion, Adherence, Guideline, Gynecological cancer

## Abstract

**Purpose:**

Receiving treatment in certified oncological centers and obtaining a second medical opinion has been proven to enhance both the quality and cost-effectiveness of care for oncological patients. Interdisciplinary care optimizes the treatment of oncological patients by validating the diagnosis and treatment recommendation, emphasizes translational research, and applies oncological therapies in a more target-oriented manner. This study aims to examine the extent of patient adherence to second medical opinions provided at the Comprehensive Cancer Center Erlangen–Metropolitan Area Nuremberg (CCC Erlangen-EMN) and investigates how specific patient characteristics such as age, gender, and type of cancer diagnosis influence the likelihood of adhering to a second opinion.

**Methods:**

This is a prospective, single-center observational study supported by the local statutory health-insurance body (*Allgemeine Ortskrankenkasse,* AOK). A total of 584 male and female patients with cancer in the fields of urology, gynecology, gastroenterology, or sarcoma, seeking a second medical opinion were assessed for their adherence to the second opinion. Levels of adherence in patient subgroups were compared using appropriate statistical tests. Correction for multiple testing was not performed, due to the exploratory nature of the study.

**Results:**

Almost 75% of the patients adhered to the second opinion recommendations and an additional 10% partially followed them. Men adhered to the second opinion recommendation slightly more often (79.1%) than women (70.7%; chi-square test, *P* = 0.01). At 83%, second-opinion adherence was highest among patients who had received an incomplete but guideline-compliant first opinion. If the first opinion was not guideline-compliant, about 67% adhered to the second opinion. Adherence to second opinions was not significantly influenced by whether the initial therapy recommendation adhered to guidelines (Fisher’s test, *P* = 0.16 for all departments, *P* = 0.27 for the gynecology department). Most patients adhered to the second opinion after primary therapy (92.9%).

**Conclusions:**

More than two-thirds of patients ultimately followed the recommendation provided in the second opinion. The results of this study enhance our understanding of patient adherence to medical advice and treatment regimens. This study demonstrated that the majority of patients adhere to second opinions and highlighted the feasibility of easy access to second opinions from a certified cancer center. Women adhered slightly less to second opinions than men. More detailed and comprehensive therapy recommendations could potentially enhance adherence rates in the future.

**Supplementary Information:**

The online version contains supplementary material available at 10.1007/s00432-025-06149-2.

## Introduction

Germany implemented a Statutory Health Insurance Modernization Act (*GKV-Modernisierungsgesetz*) in 2004, which marked the founding of the Federal Joint Committee (*Gemeinsamer Bundesausschuss,* G-BA) (Bundesanzeiger Verlag 2003). Germany’s Social Security Code, Book V (*Sozialgesetzbuch,* SGB V, Paragraph 135a) defines quality in health care in three categories: process quality, result quality, and structural quality (Social Code 2013; Wesselmann 2015). The National Cancer Plan, initiated in 2008 by the Federal Ministry of Health and other stakeholders, focuses on improving cancer screening, patient guidance, and optimizing oncological care structures (Bundesministerium für Gesundheit: Nationaler Krebsplan – Handlungsfelder 2012). Given the importance of certification within the plan and the significant demands it makes on hospitals, it is essential to conduct thorough, controlled evaluations of cancer treatment outcomes in certified centers (Schmitt et al. 2023; Schoffer et al. 2024). These frameworks laid the foundation for Germany’s cancer treatment system, with certified oncological centers that follow national and international guidelines and record quality indicators, providing transparent quality control mechanisms that are accessible to patients (Schulmeyer et al. 2023).

The process of obtaining a second medical opinion in Germany is regulated in the Strengthening Statutory Health Insurance Act (*GKV-Versorgungsstärkungsgesetz*) (Bundesanzeiger Verlag 2003), which sets out regulations regarding cost coverage by health-insurance bodies for second opinions (Bundesanzeiger Verlag 2003). The statutory health-insurance provider AOK (*Allgemeine Ortskrankenkasse*) Bavaria initiated a pilot project that allows insured individuals to seek a second medical opinion for oncological diseases through an interdisciplinary tumor board, as outlined in SGB V, § 63 paragraph 1 (Social Code 2021). Despite these advances, comprehensive data on second-opinion programs are still limited, as the programs have only emerged recently (Bruch et al. 2021).

Previous studies have reported that the percentage of patients who receive treatment in accordance with oncological guidelines is approximately 54% (Schulmeyer, et al. 2023). Unfortunately, not every patient is able to access a specialized oncological center for various reasons—e.g., a lack of knowledge or long distances to the nearest center (Hui et al. 2018). The option of obtaining a second medical opinion is now becoming increasingly important, particularly due to personalized therapy options and patients’ active involvement in researching these, particularly via the Internet (Benbassat 2019). Seeking a second opinion offers advantages, such as making additional treatment options available, and thus improves the quality and cost-effectiveness of care for oncological patients (Beckmann et al. 2009; Cheng et al. 2021). The benefits of and motivations for seeking a second opinion are a topic of ongoing research, and second opinions have been shown to avoid both overtreatment and undertreatment (Morrow et al. 2009) and to improve patient satisfaction (Loehberg et al. 2020) as a result of the added value they provide in diagnosis and/or therapy (Mellink et al. 2006). Research indicates that about 70% of patients seek more information and/or reassurance after receiving an initial therapy recommendation (Tattersall et al. 2009). There is also a significant correlation between seeking a second opinion and patients’ disease-related fear and distress (Krebs et al. 2019).

Adherence to guidelines is an important outcome-determining factor and is also associated with survival benefits (Schwentner et al. 2013; Hébert-Croteau et al. 2004). Numerous national and international guidelines are available on diagnosis, treatment, and follow-up for most cancer entities. Adhering to evidence-based guidelines and receiving treatment in certified centers has been linked to improved outcomes and better overall survival for patients (Schulmeyer, et al. 2023; Schwentner et al. 2013; Graham and Harrison 2005; Wockel, et al. 2017; Vernooij et al. 2007). For example, a significantly higher overall survival after 5 years has been reported for breast cancer patients (HR 0.77 [0.74; 0.81]). While most published data focus on guideline adherence for physicians or patients’ adherence to medication (Krege and Goebell 2019; Panahi et al. 2022; Baryakova et al. 2023), there is a dearth of studies addressing the complexity of patient adherence and nonadherence to overall treatment plans.

The aims of this prospective study were to analyze whether cancer patients who seek a second opinion adhere to the second opinion, and to identify characteristics of patients treated in gynecological, urological, or surgical departments that may influence adherence to the second opinion.

## Methods

**Fig. 1:**
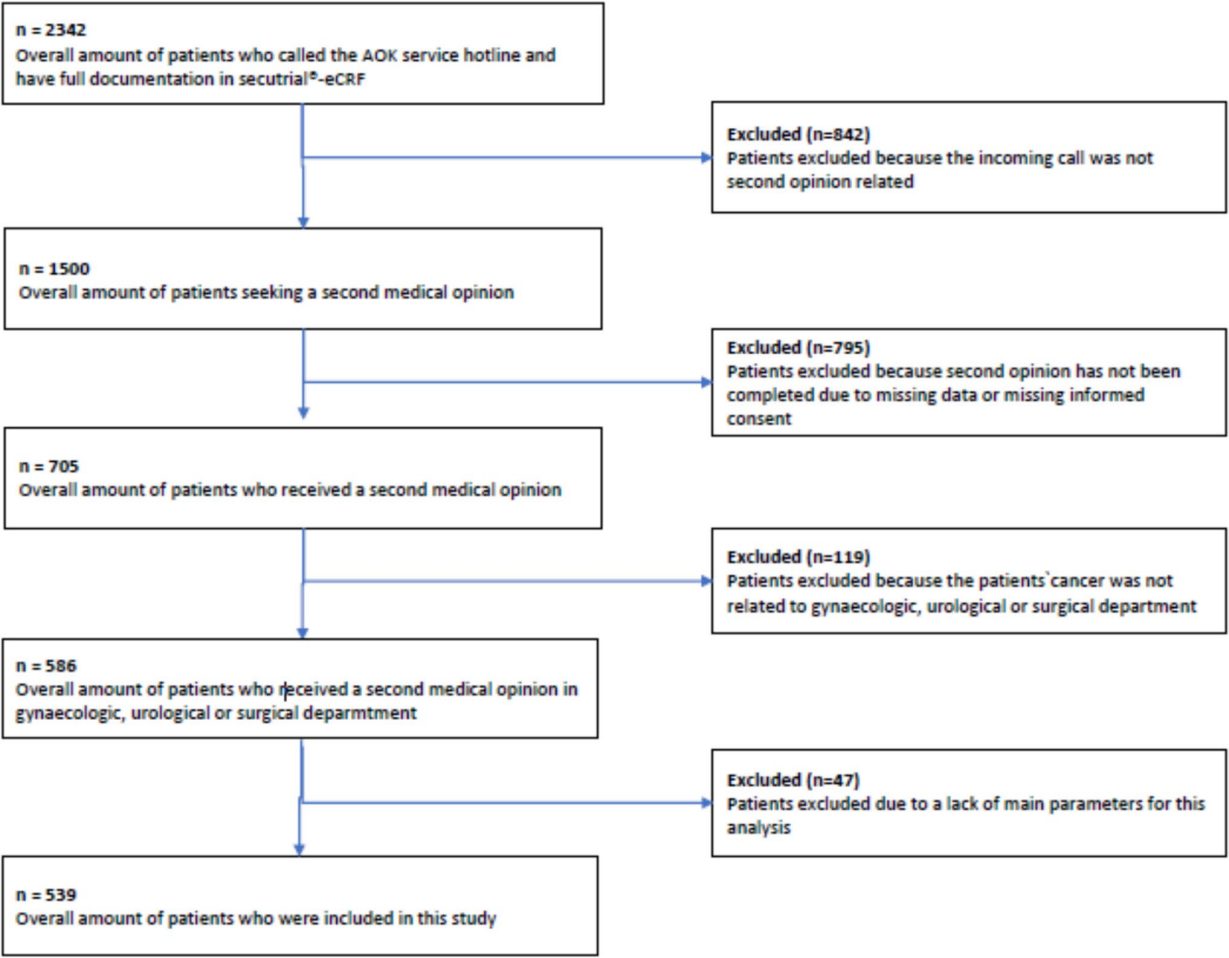
Flowchart for patient selection

Patient enrollment took place between 1 June 2014 and 31 May 2020. The final analysis included 539 patients who met the eligibility criteria (Fig. 1). Eligible patients included men and women who called the AOK (statutory health insurance) service number to reach the cancer information service at Erlangen University Hospital and received a second opinion regarding their cancer therapy. Cancer patients in the fields of urology, gynecology, gastroenterology, and sarcomas were included. Exclusion criteria encompassed patients who contacted the service number for reasons unrelated to seeking a second oncological opinion, patients who did not provide their full cancer history, patients with malignancies other than in the fields of urology, gynecology, gastroenterology, or sarcoma, and patients who had had outpatient interactions with the hospital within the preceding 3 months.

The amount of contact time was documented in minutes. If there were multiple periods of contact, the overall amount of time spent was added up and termed “total time spent.”

### Assessment of guideline compliance

For all study participants with malignancies in the fields of urology, gynecology, gastroenterology, or sarcoma (n = 539), the following patient characteristics were collected: age in years, gender, tumor status, and therapy status. The guideline compliance of the initial therapy recommendation was also noted (initial opinion complete and compliant with guidelines; first opinion guideline-compliant, but incomplete; first opinion not in line with guidelines; and not evaluable/missing). The patients were deemed “not evaluable” if they met one of the three following criteria: “first opinion cannot be assessed / missing data,” “no guideline available for tumor entity,” or “no information / tumor entity not part of the evaluation.” These patients were excluded from the analysis for group differences.

### Assessment of adherence

*Second-opinion adherence for patients in the overall population.* For all study participants with malignancies in the fields of urology, gynecology, gastroenterology, or sarcoma (n = 539), second-opinion adherence was classified into three groups: “yes,” “partial,” and “no adherence.” Adherence was defined as following the second-opinion therapy recommendations. Partial adherence was determined if patients did not adhere to all of the therapy recommendations. No adherence was defined as not following the second-opinion therapy recommendations at all.

*Detailed second-opinion adherence for patients in the department of gynecology.* For these patients (n = 217), second-opinion adherence was classified into four groups: “yes,” “partial/core elements not violated,” “partial/core elements violated,” and “no.” In partial adherence, a further evaluation was conducted to determine whether core elements of guideline-based treatment recommendations were adhered to or violated. Accordingly, patients with partial second-opinion adherence were classified into the two groups: “partial/core elements not violated” and “partial/core elements violated.” The core elements and noncore elements are listed in Supplement 1.

### Study design

All patients who contacted the cancer information service affiliated with the Comprehensive Cancer Center (CCC) for the Erlangen–Nuremberg metropolitan area via the AOK service number were systematically documented.

The detailed procedure involved in tumor board recommendations and evaluating the guideline compliance of the first opinion is described in detail elsewhere (Schulmeyer, et al. 2023). In brief, after written consent had been obtained from the patients, their cases with the first oncological therapy recommendation were presented to the interdisciplinary tumor board. The tumor board’s recommendations were documented on a second-opinion form. Whether the first opinion’s treatment recommendation complied with national guidelines were evaluated by two medical experts into the following groups: guideline-compliant and complete; guideline-compliant, but incomplete; not in line with guidelines. In the event of discrepancies between these two, the decision of the more experienced assessor was used based on his or her clinical experience. A summary document detailing the tumor board’s recommendations, complying with national guidelines, was provided to the patients. The patients had an opportunity to contact the Comprehensive Cancer Center again for any further questions or clarifications.

Three months later, follow-up inquiries to the patients were conducted concerning the current treatment status. If this information was not assessable, the reason for it was identified and documented (e.g., because the patient had died or had withdrawn consent). On the basis of the information gathered, the extent of the patient’s adherence to the recommendations offered in the second opinion was evaluated.

### Ethics

The study received approval from the Ethics Committee at Erlangen University Hospital (no. 175_13B) and was conducted in accordance with the ethical principles of the Declaration of Helsinki.

### Statistical analysis

The association between patient characteristics and second-opinion adherence (categorical; “yes,” “partial,” “no”) was tested statistically in each case. Patients for whom adherence could not be assessed or information on adherence was missing were excluded from the analysis. For categorical characteristics, a chi-square test or Fisher’s exact test was used. A chi-square test was used if all the expected cell frequencies were greater than 5; otherwise, Fisher’s exact test was used. For the continuous characteristic of patient age, an analysis of variance (ANOVA) was performed if the residuals were normally distributed and variance homogeneity was present. If this was not the case, a Kruskal–Wallis test was used.

In addition, a sensitivity analysis was carried out. The variable “second-opinion adherence” was taken as an ordinal-categorical variable. For categorical patient characteristics, an ordinal chi-square test was performed in each case. For ordinal-categorical or continuous characteristics, Spearman’s correlation coefficient ρ was calculated in each case.

The joint influence of patient characteristics on second-opinion adherence was investigated using a multivariable, multinomial logistic regression model. The outcome variable was second-opinion adherence (“yes,” “partial,” and as a reference category “no”). As information regarding the concordance of the first and second therapy recommendations was only available for part of the study population, one regression model with this variable and another without it were set up. Patients with missing observations were excluded from the analysis. Adjusted odds ratios with associated 95% confidence intervals were estimated for each predictor.

The associations between patient characteristics and the outcome “total time spent” were explored using univariable negative binomial regression analyses. The joint influence of patient characteristics on “total time spent” was analyzed using a multivariable negative binomial regression model.

## Results

### Patient group

Patient recruitment took place between June 2014 and May 2020. A total of 2342 patients contacted the Bavarian AOK service number. Among them, 1500 patients were seeking information about a second opinion; the remaining calls were made for various reasons—e.g., address changes or loss of the patient’s insurance card. A total of 705 patients received a second opinion. The remaining patients did not receive a second opinion (because no documents were submitted, for example). Among these 705 patients, 586 were from departments participating in the analysis—gynecology, urology, and surgery. Among these, 47 patients were excluded due to missing information in the main variables. A flowchart of the patient recruitment process is shown in Fig. 1.

For the department of gynecology, with 232 patients, 15 patients for whom second-opinion adherence was “not assessable” (e.g., because the patient had died or had withdrawn consent) were excluded. A total of 217 patients were thus included in the analysis of the gynecology department.

### Patient characteristics

The mean age of the 539 patients was 60.8 years (standard deviation, SD 12.4). Among those who adhered to the treatment regimen, the mean age was 58.2 years (SD 13.1) for partial adherence and 62.3 years (SD 12.4) for those with no adherence. Overall, 57.7% of the patients were female (n = 311) and 41.7% were male (n = 225). The primary diagnosis was without distant metastases in 34.3% of cases (n = 185) and with distant metastases in 37.7% (n = 203). The patients were tumor-free in 19.3% of cases (n = 104). Most patients had not received treatment at the time of consultation, but were already planning therapy in 67.7% of cases (n = 365). The patients were receiving primary therapy in 17.6% of cases (n = 95) and palliative therapy in 12.1% (n = 65; Table 1). The patient characteristics—classified into adherence, partial adherence, and no adherence—are shown in Table 1.

**Table 1:** Patient characteristics relative to second-opinion adherence status (n = 539)

Characteristic	Category	Second-opinion adherence^1^ (n = 539)			*P* value
		Yes (n = 401)	Partially (n = 52)	No (n = 86)	
Age (years)	Median (IQR)	61 (53–70)	57 (50–69)	63 (54–70)	0.175 (Kruskal–Wallis)
	Mean (SD)	60.8 (12.4)	58.2 (13.1)	62.3 (12.4)	
	Missing values	0	0	0	
Gender	Female	220 (70.7)	40 (12.9)	51 (16.4)	0.012 (chi-squared)
	Male	178 (79.1)	12 (5.3)	35 (15.6)	
	Missing values	3	0	0	
Tumor status^2^	Tumor-free	73 (70.2)	14 (13.5)	17 (16.3)	0.308 (Fisher)
	Primary tumor	143 (77.3)	13 (7.0)	29 (15.7)	
	Distant metastases	149 (73.4)	24 (11.8)	30 (14.8)	
	Recurrence	27 (75.0)	1 (2.8)	8 (22.2)	
	Missing values	9	0	2	
Therapy status	No therapy / therapy planned	275 (75.3)	32 (8.8)	58 (15.9)	0.469 (Fisher)
	Receiving primary therapy	66 (69.5)	13 (13.7)	16 (16.8)	
	After primary therapy	13 (92.9)	1 (7.1)	0 (0)	
	Palliative therapy	47 (72.3)	6 (9.2)	12 (18.5)	
	Missing values	0	0	0	
Guideline compliance of initial therapy recommendation	Initial opinion complete and compliant with guidelines	213 (72.0)	37 (12.5)	46 (15.5)	0.162 (Fisher)
	First opinion guideline-compliant, but incomplete	59 (83.1)	3 (4.2)	9 (12.7)	
	First opinion not in line with guidelines	24 (66.7)	4 (11.1)	8 (22.2)	
	Not evaluable/missing^3^	105	8	23	

*IQR* interquartile range, *SD* standard deviation

^1^Absolute and percentage frequencies for all characteristics except age

^2^The original categories were combined as follows: primary tumor (suspected tumor, primary tumor), distant metastases (primary tumor/distant metastases, distant metastases), recurrence (suspected recurrence, recurrence)

^3^Here, the original categories “first opinion cannot be assessed/missing data,” “no guideline available for tumor entity,” and “no information/tumor entity not part of the evaluation” were combined

The mean ages of the 217 female patients from the gynecology department were 55 years for those who adhered to the treatment regimen and 57 years for those with no adherence. Approximately equal numbers of patients were tumor-free (30.9%, n = 67), had a primary tumor (28.1%, n = 61) and had distant metastases (30%, n = 65).

Most patients had not received treatment at the time of consultation, and were planning therapy in 64.1% of cases (n = 139; Table 2). Table 2 also lists the characteristics of the gynecology patients, classified into adherence, partial adherence, and no adherence.

**Table 2:** Detailed second-opinion adherence among patients from the gynecology department (n = 217)

Characteristic	Category	Second-opinion adherence^1^ (n = 217)				*P* value
		Yes (n = 145)	Partial/core elements not violated (n = 14)	Partial/core elements violated (n = 25)	No (n = 33)	
Age (years)	Median (IQR)	55 (47–66)	56 (18–63)	54 (45–63)	57 (52–66)	0.648 (Kruskal–Wallis)
	Mean (SD)	56.9 (12.9)	54.9 (13.1)	54.8 (12.5)	57.6 (12.8)	–
	Missing values	0	0	0	0	–
Tumor status^2^	Tumor-free	41 (61.2)	5 (7.5)	8 (11.9)	13 (19.4)	0.454 (Fisher)
	Primary tumor	45 (73.8)	3 (4.9)	7 (11.5)	6 (9.8)	
	Distant metastases	42 (64.6)	6 (9.2)	9 (13.8)	8 (12.3)	
	Recurrence	11 (61.1)	0 (0.0)	1 (5.6)	6 (33.3)	
	Missing values	6	0	0	0	–
Therapy status	No therapy/therapy planned	93 (66.9)	9 (6.5)	16 (11.5)	21 (15.1)	0.932 (Fisher)
	Receiving primary therapy	38 (66.7)	3 (5.3)	7 (12.3)	9 (15.8)	
	After primary therapy	2 (66.7)	0 (0.0)	1 (33.3)	0 (0.0)	
	Palliative therapy	12 (66.7)	2 (11.1)	1 (5.6)	3 (16.7)	
	Missing values	0	0	0	0	–
Guideline compliance of initial therapy recommendation (fewer categories)	Initial opinion complete and compliant with guidelines	99 (66.4)	9 (6.0)	19 (12.8)	22 (14.8)	0.269 (Fisher)
	First opinion guideline-compliant, but incomplete	12 (70.6)	0 (0.0)	2 (11.8)	3 (17.6)	
	First opinion not in line with guidelines	9 (64.3)	1 (7.1)	2 (14.3)	2 (14.3)	

*IQR* interquartile range, *SD* standard deviation

^1^Absolute and percentage frequencies for all characteristics except age

^2^The original categories were combined as follows: primary tumor (suspected tumor, primary tumor), distant metastases (primary tumor/distant metastases, distant metastases), recurrence (suspected recurrence, recurrence)

### Second-opinion adherence

Among the overall group of 584 patients, 68.7% adhered to the second opinion (Table 3). In addition, 8.9% partially implemented the second opinion.

**Table 3:** Second-opinion adherence (n = 584)

Second-opinion adherence	n	%
Patient has adhered to the second opinion	401	68.7
Patient has partially complied with the second opinion	52	8.9
Patient did not comply with the second opinion	86	14.7
Not assessable	45	7.7

Among the 217 patients from the gynecology department, 145 (66.8%) adhered to the second opinion (Table 2). Among patients who partially implemented the second opinion, a further evaluation was conducted to determine whether core elements of the guideline-based therapy recommendations were adhered to or violated. Among the 39 patients from the gynecology department who had partial adherence to the second opinion, core elements of a guideline-based therapy recommendation were violated in 25 women (64.1%). The remaining 14 women carried out the core elements and disregarded other recommended measures. Core elements and noncore elements are listed in Supplement 1.

### Associations between patient characteristics and second-opinion adherence (univariable analysis)

**Fig. 2:**
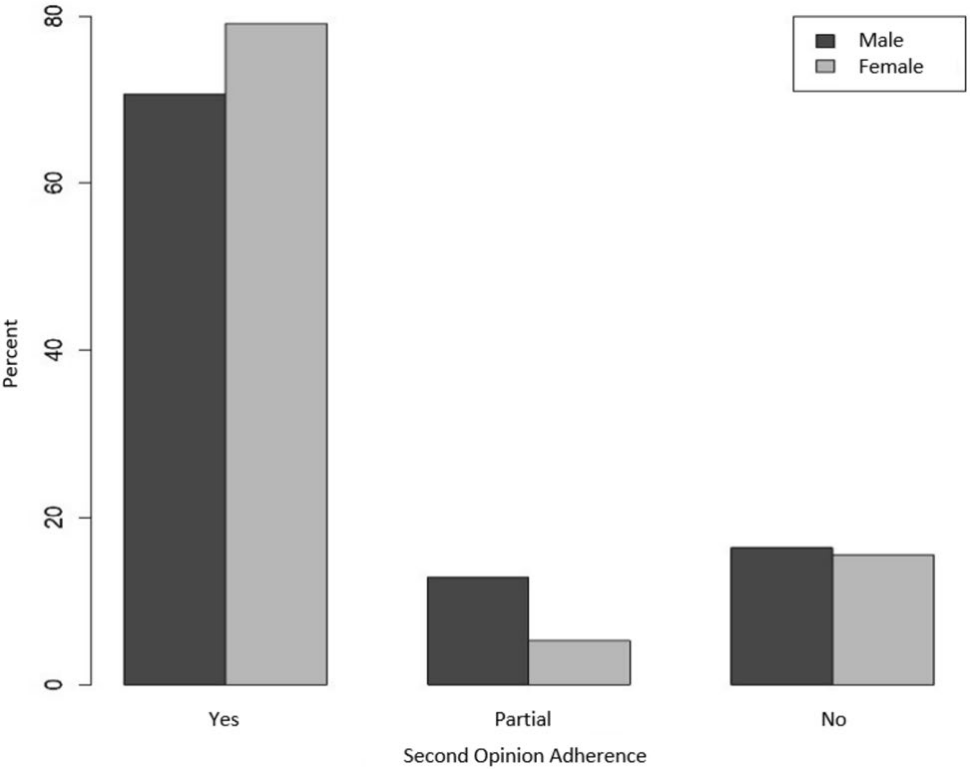
Frequency of adherence relative to gender, across categories (%)

The only significant group differences for patient characteristics were for gender (*P* = 0.01). Men showed slightly better second-opinion adherence (79.1%) than women (70.7%) (Table 4; Fig. 2). Women complied only partially with the second-opinion recommendation twice as often (12.9%) as men (5.3%). The proportions of men and women who did not adhere to the second opinion were roughly equivalent, at around 16%. Figure 2 shows the percentage frequencies of adherence relative to gender for each category.

**Table 4:** Odds ratios (95% confidence intervals) for second-opinion adherence (n = 525 complete observations)

Characteristic	Category	Second-opinion adherence	
		Yes vs. no^1^	Partially vs. no^1^
Gender	Female	Reference	Reference
	Male	1.32 (0.71–2.45)	0.55 (0.21–1.44)
Age (y)		0.99 (0.97–1.01)	0.99 (0.96–1.02)
Tumor status^2^	Tumor-free	Reference	Reference
	Primary tumor	1.07 (0.44–2.60)	1.09 (0.31–3.86)
	Distant metastases	1.09 (0.44–2.74)	1.84 (0.52–6.53)
	Recurrence	0.77 (0.24–2.47)	0.31 (0.03–3.21)
Therapy status	No therapy / therapy planned	Reference	Reference
	Receiving or after primary therapy	1.57 (0.63–3.91)	2.45 (0.74–8.10)
	Palliative therapy	0.73 (0.31–1.69)	0.84 (0.25–2.81)

^1^The category “no” is the reference category for odds ratios

^2^The original categories were combined as follows: primary tumor (suspected tumor, primary tumor), distant metastases (primary tumor/distant metastases, distant metastases), recurrence (suspected recurrence, recurrence)

There were no significant differences in age between the three groups (Kruskal–Wallis test, *P* = 0.18). Patients who partially adhered to the second opinion were slightly younger (mean age 58.2 years) than patients in the other two groups (mean age 60.8 years for adherence and 62.3 years for no adherence; Table 1).

Analysis of the tumor status showed that the proportion of patients who adhered to the second opinion was higher among patients with a suspected or primary tumor (77.3%) than in the other groups, without statistical significance (*P* = 0.31). Additionally, there were no significant group differences in second-opinion adherence for therapy status (*P* = 0.47; Table 1). Among patients who had not started therapy or scheduled therapy at the time of assessment, 75.3% adhered to the second-opinion recommendation.

### Associations between patient characteristics and second-opinion adherence in patients from the gynecology department (univariable analysis)

Among patients from the gynecology department, there were no significant differences in age between the four adherence groups (*P* = 0.65). Patients who did not adhere to the second opinion were the oldest, at 57 years (Table 2).

There were no associations between tumor status and second-opinion adherence (*P* = 0.45). Patients with a primary tumor showed the highest level of adherence to the second opinion (73.8%; Table 2). Relative to treatment status, no significant differences in second-opinion adherence were observed between the groups (*P* = 0.93). Across all treatment status groups, approximately 67% of patients adhered to the second-opinion recommendation.

The extent to which the initial therapy recommendation aligned with the guidelines did not significantly influence second-opinion adherence (*P* = 0.27; Table 2). At approximately 71%, second-opinion adherence was highest among patients who had a first opinion that was not completely guideline-compliant. If the first opinion was not guideline-compliant, the proportion was about 64%. In addition, if the first and second treatment recommendations agreed, the majority of patients adhered to the second opinion (66.4%; Table 2). This proportion was lower, at 59%, when the recommendations did not match. Overall, however, no significant associations were observed (*P* = 0.09).

### Guideline compliance of the first opinion

The extent to which the initial therapy recommendation was in conformity with guidelines did not exert a significant influence on adherence to the second opinion (Fisher’s exact test, *P* = 0.16; Table 1). Among patients whose initial opinion was guideline-compliant but incomplete, adherence to the second opinion reached 83%. When the initial opinion was not guideline-compliant, adherence to the second opinion was approximately 67%.

The sensitivity analyses, in which categorical variables were taken ordinally where appropriate and the variable “age” was used continuously, produced results similar to the main analysis. Second-opinion adherence was significantly higher in women than in men (*P* = 0.01). No significant correlation with second-opinion adherence was observed for any other patient characteristics.

### Associations between patient characteristics and second-opinion adherence (multivariable analysis)

This analysis included all observations from the three departments analyzed that had complete data (n = 525).

Due to the large number of missing values (approximately 25%) for the variable “guideline compliance of initial therapy recommendation,” it was excluded from the model. In addition, the categories of “Receiving primary therapy” and “After primary therapy” were combined due to small case numbers.

None of the variables in the multinomial, multivariable regression model showed an odds ratio significantly different from 1 (Table 4).

Using a separate model for the patients from the gynecology department, including the variable “agreement between first and second therapy recommendation,” was not feasible due to the small number of cases (Table 2).

### Associations between patient characteristics and total time spent

**Fig. 3:**
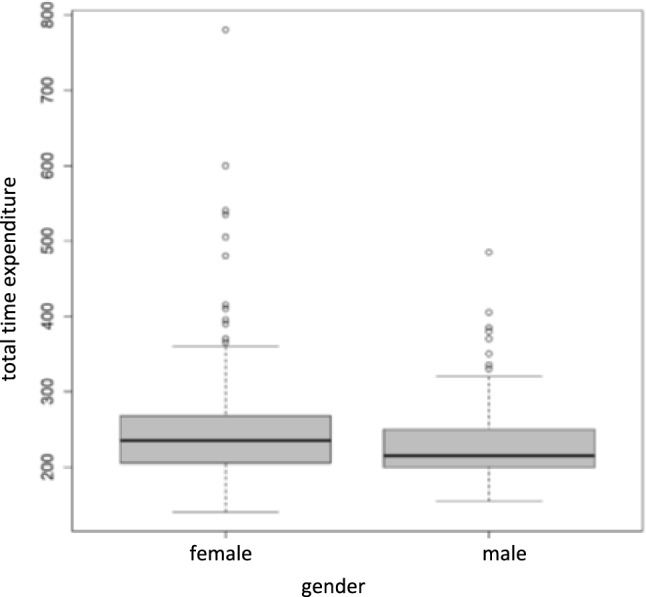
Box plot for the total time required to formulate a second opinion in minutes, relative to gender. The median times for woman and men were 235 min (IQR 205.0–267.5 min) and 215 min (IQR 200–250 min), respectively

A median of 20 min more was needed to formulate the second opinion for women (235 min) than for men (215 min; Table 5; see Fig. 3 for the distribution of the total time required for the two sexes in a box plot). Time expenditure was thus significantly higher in women than in men (*P* < 0.001). With increasing patient age, the time needed to formulate the second opinion decreased (Table 5). Tumor status also had a significant effect on time spent (*P* = 0.04). In patients with distant metastases or recurrences, the time spent was higher than in tumor-free patients or patients with a primary tumor. No significant correlations were observed between the other patient characteristics investigated and time spent. The univariable associations between patient characteristics and time spent were confirmed with a multivariable negative binomial regression analysis (Table 6). Both gender and tumor status had a significant influence on the time spent.

**Table 5:** Influence of patient characteristics on total time to second opinion in minutes (n = 584). Median and interquartile range (IQR) are descriptive evaluations. Expected values and *P* values are from univariate negative binomial regression analyses

Characteristic	Category	n	Median (minutes)	IQA (minutes)	Time expenditure (mean, 95% CI)	*P* value
Gender	Female	343	235	205–267.5	246.4 (240.7–252.3)	< 0.001
	Male	238	215	200–250	230.0 (223.6–236.6)	
Age	< 53 years	139	235	210–255	243.2 (234.3–252.5)	0.66
	53–70 years	293	225	200–260	239.3 (233.3–245.6)	
	≥ 70 years	152	220	200–256.2	237.6 (229.2–246.2)	
Tumor status^1^	Tumor-free	106	225	205–250	237.2 (227.4–247.4)	0.04
	Primary tumor	194	225	200–258.8	234.1 (226.9–241.5)	
	Distant metastases	234	230	205–273.8	245.9 (239.1–253.0)	
	Recurrence	37	235	205–265	254.5 (237.1–273.2)	
Therapy status	No therapy / therapy planned	392	225	203.8–260	239.9 (234.6–245.3)	0.94
	Receiving primary therapy	99	225	200–260	241.3 (230.8–252.2)	
	After primary therapy	15	220	197.5–260	244.1 (218.0–273.5)	
	Palliative therapy	78	225	205–263.8	236.8 (225.3–248.9)	
Guideline compliance of initial therapy recommendation	Initial opinion complete and compliant with guidelines	318	227.5	205–265	242.2 (236.5–248.0)	0.11
	First opinion guideline-compliant, but incomplete	77	220	200–250	229.3 (218.5–240.6)	
	First opinion not in line with guidelines	40	225	200–255	233.8 (218.7–249.9)	

^1^The original categories were combined as follows: primary tumor (suspected tumor, primary tumor), distant metastases (primary tumor/distant metastases, distant metastases), recurrence (suspected recurrence, recurrence)

**Table 6:** Adjusted mean total time to second-opinion formulation with 95% confidence intervals (95% CI; n = 568 complete observations)

Characteristic	Category	Time expenditure	*P* value
Gender	Female	249.2 (241.2–257.3)	< 0.001
	Male	232.3 (223.9–241.0)	
Age	< 53 years	240.8 (230.4–251.6)	0.93
	53–70 years	239.5 (231.7–247.6)	
	≥ 70 years	241.5 (231.6–251.8)	
Tumor status^1^	Tumor-free	229.1 (218.1–240.5)	0.01
	Primary tumor	234.2 (225.5–243.2)	
	Distant metastases	246.3 (238.3–254.4)	
	Recurrence	253.6 (235.6–273.0)	
Therapy status	No therapy / therapy planned	239.8 (233.2–246.7)	0.43
	Receiving or after primary therapy^2^	246.7 (235.4–258.6)	
	Palliative therapy	235.3 (221.8–249.6)	

^1^The original categories were combined as follows: primary tumor (suspected tumor, primary tumor), distant metastases (primary tumor/distant metastases, distant metastases), recurrence (suspected recurrence, recurrence)

^2^Due to the small number of cases in the category “after primary therapy” (n = 15), this category and the category “receiving primary therapy” were combined into one

## Discussion

The aim of this prospective study was to enhance the quality and effectiveness of care for cancer patients seeking a second opinion, particularly in surgical, gynecological, and urological departments. It examined the influence of patient characteristics on second-opinion adherence. Following the formulation of the second opinion, more than two-thirds of patients adhered to its recommendations. This AOK study comprises the largest cohort of cancer patients in Germany to date, emphasizing the dynamics of patients’ adherence to treatment recommendations.

In this study, the level of adherence among the overall group was almost 10% higher for men than women. This is consistent with current data showing that men adhere to recommendations more often than women (Venditti, et al. 2023; Rebic et al. 2023), and provides an important insight into gender-based medicine. However, this statement cannot be transferred to the group from the gynecology department, as more women than men were present there.

There were no significant correlations between the guideline compliance of the first opinion and adherence to the second opinion. However, most patients adhered to the second opinion if it was consistent with the first opinion. With regard to adherence, partial adherence, and nonadherence, there were no significant differences in age, tumor status, therapy status, or whether the first therapy recommendation was guideline-compliant. However, it was noted that partial adherence was more common among younger patients than in the other groups.

Interestingly, the level of adherence was independent of whether or not the initial opinion was guideline-compliant. The study showed that adherence was greater when the initial recommendation was at least partially guideline-compliant. However, patients are not able to assess, or can only partially assess, the guideline compliance of their recommendations. Factors that motivate patients to make the effort to seek multiple oncological opinions and then not adhere to them have yet to be identified.

However, one lever for improving adherence that could be identified was the provision of a more detailed and comprehensive document with therapy recommendations. The existing forms provided to patients are brief and concise, geared more towards health-care professionals. The patient then relies on explanations from other doctors or has to actively contact the Comprehensive Cancer Center again. This could be actively redesigned in the future in order to increase adherence.

It has been shown that interdisciplinary tumor conferences improve the quality of patient care, treatment plans, and therefore overall survival (Kesson et al. 2012; Farrugia et al. 2015; Newman et al. 2006). In addition, treatment in centers certified by the German Cancer Society (*Deutsche Krebsgesellschaft,* DKG) has been shown to improve health outcomes and even overall survival among oncological patients (Schmitt et al. 2023; Kowalski et al. 2015; Wesselmann et al. 2014). This study discusses an easily assessable, patient-initiated option for seeking a second opinion, breaking down barriers for oncological patients.

### Strengths and limitations

To the best of our knowledge, this study includes the largest cohort of patients to investigate adherence after seeking second opinions in Germany, encompassing diverse tumor types across multiple oncological centers.

Limitations of the study include the limited comparability of this patient group with others in similar studies, as all patients were from the same insurance class and were predominantly in good health (Mindell et al. 2015). The patient-initiated nature of the second opinions excludes certain cohorts—e.g., those with poorer health status—despite their potential benefit from such consultations. While demographic factors may influence second-opinion patterns and outcomes, this study provides valuable insights into patients’ adherence in cancer treatment. However, the lack of detailed pathological and clinical descriptions, as well as the heterogeneous group and the undifferentiated assessment of female and male patients in the group, is acknowledged as a limitation, since the focus was on broader patterns rather than specific pathological nuances. Moreover, patients’ motivations for nonadherence were not identified.

## Conclusion

The results of this study may contribute to a better understanding of patient adherence to medical advice and treatment regimens. The study showed that a majority of the patients adhere to the second opinion, and it demonstrated the feasibility of providing easy access to a second opinion by a certified cancer center. This research area—examining guideline adherence, second-opinion adherence, the effects of certified oncological centers and their cost-effectiveness—is an important part of health-care research. It has improved patient care in the past, and also in this study. This should be used for reassessment of the structural development of health care in Germany in the future. The extent of nonadherence was similar across the overall population, showing that there are other factors that influencing patients’ treatment adherence. Further research is needed to identify other factors in order to optimize patients’ adherence and thus their overall survival.

## Supplementary Information

Below is the link to the electronic supplementary material.

Supplementary file1 (DOCX 16 KB)

## Data Availability

The datasets are available from the corresponding authors upon reasonable request.

## Abbreviations

ANOVA: Analysis of variance
AOK: *Allgemeine Ortskrankenkasse* (Statutory health-insurance body)
CCC: Comprehensive Cancer Center
DKG: *Deutsche Krebsgesellschaft* (German Cancer Society)
HR: Hazard ratio
SD: Standard deviation
SGB: *Sozialgesetzbuch* (Social security code)
