# Supplementary material for: Improving quality of care for cancer patients through oncological second opinions in a Comprehensive Cancer Center: adherence to second-opinion therapy recommendations
Source: J Cancer Res Clin Oncol. 2025 Apr 2;151(4):130. doi: 10.1007/s00432-025-06149-2 (PMC11965187; doi:10.1007/s00432-025-06149-2)
Supplement: Supplementary file 1 — Supplementary file1 (DOCX 16 KB) [file 432_2025_6149_MOESM1_ESM.docx]

# Supplement 1

Core elements for partial adherence among patients from the gynecology department (n = 217).

| Core elements violated | Core elements not violated |
| --- | --- |
| Surgery | Further imaging |
| Radiotherapy | Genetic testing |
| Chemotherapy | Bone-specific therapy |
| Endocrine therapy |  |
